# COGcollator 2.0: A Tool for Analyzing Distant Evolutionary Relationships Among Families of Homologous Proteins

**DOI:** 10.32607/actanaturae.27789

**Published:** 2026

**Authors:** D. V. Dibrova, S. Y. Rykov

**Affiliations:** Lomonosov Moscow State University, Belozersky Institute of Physico-Chemical Biology, Moscow, 119234 Russia; Lomonosov Moscow State University, School of Bioengineering and Bioinformatics, Moscow, 119234 Russia; Institute of Philosophy, Russian Academy of Sciences, Moscow, 109240 Russia

**Keywords:** clusters of orthologous groups, COG, phylogenomic analysis, web server, protein domain structure

## Abstract

The analysis of protein evolutionary relationships is of both theoretical and practical significance. Identifying and characterizing evolutionary relationships between the components of protein complexes and metabolic enzymes fosters a deeper understanding of their evolutionary history and molecular evolution. The existence of related enzymes makes the annotation of new sequences complicated, since this process relies heavily on accurately determining the protein family classification of the sequence being analyzed. Clusters of Orthologous Groups (COGs) are widely used for the classification of prokaryotic proteins. COGs are constructed based on the occurrence of the respective protein-coding genes within complete genomes. Previously, we introduced COGcollator, a tool designed to visualize the relatedness between COGs by analyzing the hits of their profile HMMs (Hidden Markov Models). This paper presents an update of the COGcollator web service. It is based on the latest version of the COG database and features a completely new interface and additional functionalities. To demonstrate the capabilities of our tool and the validity of the data, we present the COGcollator results for the subunits of NADH:quinone oxidoreductase type 1 (NDH-1), a homologue of the mitochondrial complex I, as the evolutionary relationships of NDH-1 with other protein complexes have been extensively documented in the literature. The web service is available free of charge without registration at https://boabio.belozersky.msu.ru/en/COGcollator. Through the web service interface, users can access pre-calculated COGcollator results for 4,972 COGs and download their respective profile HMMs.

## INTRODUCTION

Functional analysis of an amino acid sequence begins by searching for similarities to sequences with known functions. Non-random similarities between proteins suggest homology, i.e. a shared ancestral sequence, between their corresponding genes. However, it does not clarify the extent to which the functional annotation of one protein is applicable to its homologue. There are two primary types of homology relationships between proteins and their genes [1]: they can be either orthologs, that is, proteins whose ancestral gene sequences diverged due to speciation (such proteins often perform the same function), or paralogs, which arise as a result of gene duplication (they, in contrast, typically exhibit functional divergence). Such a classification, however, cannot always be directly applied to the genes of a specific genome, given the often case when a gene is formed by the fusion of several parts. Orthologous genes are commonly identified across the genomes of distinct species. In contrast, paralogous genes can reside within the genome of a single species. However, due to frequent gene loss events throughout evolution and horizontal gene transfer [2, 3], this is not always the case. Clusters of Orthologous Groups (COGs) have been created to enhance the accuracy of protein functional classification. The strategy based on COGs combined an analysis of gene occurrence across diverse genomes with sequence comparison [4]. The process of COG creation and their annotation have been considerably refined over the past 20 years [5, 6, 7], and the COG database has received an update in the last year [8]. While other tools, including eggNOG [9], are also based on the deduction of orthologous protein groups, the KEGG metabolic pathway database maps orthologous groups specifically to COGs [10, 11].

**Fig. 1:**
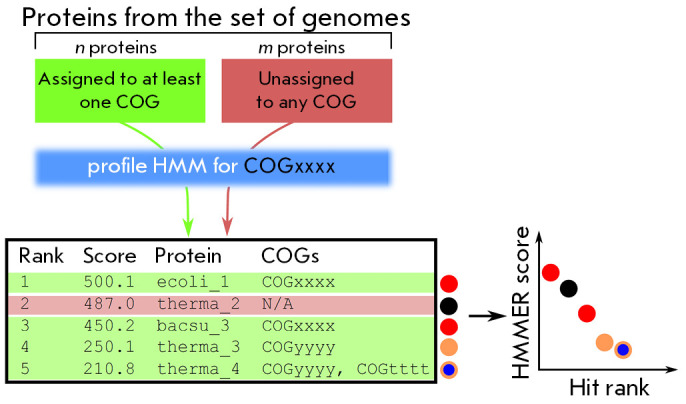
The general idea behind the COGcollator 2.0 approach is based on analyzing the hits list of a profile HMM for a specific COG. The list of hits from the profile HMM is visualized as a function of the alignment score versus the hit rank. The assignment of each protein to specific COGs, based on the information available in the COG database, is depicted with different colors

Previously, we developed COGcollator, a tool designed to investigate the evolutionary relationships between distinct COGs [12]. The idea behind our approach is to align a mathematical model (a profile HMM) of a specific COG both with proteins assigned to COGs and with the remaining unassigned proteins from the same genomes (Fig. 1). The search result of a profile HMM for a COG among all proteins in the selected set of genomes can be represented as a graph. In this graph, each point corresponds to a single protein, with the X-axis indicating the hit rank (the sequential position of the hit within the list of all profile hits, sorted by alignment score), the Y-axis denoting the corresponding alignment score, and the colors of the points indicating the COG assignments of each protein. If a substantial number of proteins assigned to other COGs are found among the significant (i.e., high-scoring) hits of a given profile HMM, this may indicate a homologous relationship between them. Furthermore, the visual representation of this graph can elucidate the degree of relatedness among protein families and lead to unexpected conclusions. For instance, the graphs for the catalytic and noncatalytic subunits of F- and A-type rotary membrane ATP synthases (COG0055/COG0056 and COG1155/ COG1156) demonstrate that all of these subunits are far more similar to the flagellar ATPase subunit FliI (COG1157) than to each other. Explaining this observation is challenging, assuming that the subunit pairs α/B and β/A do represent ancient paralogues originating from gene duplication preceding the divergence of the last common archaeal and bacterial ancestor [13].

The COGcollator tool required a significant update due to three primary factors. First, a new version of the COG database was published in 2021, which doubled the number of prokaryotic genomes compared to the 2015 version used in our previous study (1,309 genomes versus 711) and introduced over 250 new COGs. These additional COGs included 118 components of photosystems and other photosynthesis-related COGs, 125 COGs associated with sporulation processes, and 21 COGs describing CRISPR-Cas system proteins. The latest update to the COG database, published in 2024, expanded the COG number further by 104 COGs mostly covering various types of prokaryotic secretion systems, and nearly doubled the number of incorporated organisms to 2,296, now covering previously unrepresented taxa such as Archaea from the Asgard phylum (Promethearchaeota) [8]. In addition, only 4,534 out of 4,631 COGs were considered in our first study due to their small size. Second, subsequent analysis revealed that approximately 5% of the constructed mathematical models exhibited low sensitivity, failing to identify at least 20% of the proteins designated for a specific COG in the original COG database. These profiles required revisions. Third, new functionalities had to be added into the software in order to enhance user-friendliness and improve the clarity of the outputs.

In this study, we present a revised version of our COGcollator tool featuring a new web interface compatible with the latest COG database, along with corrected mathematical models for identifying new COG members and a number of novel models. Additionally, several key features are included in the tool, as described further below. The user interface includes comprehensive descriptions for all options and is now available in both the Russian (default) and English languages.

## EXPERIMENTAL

**Constructing profile HMMs for COGs**

*General principle for constructing profile HMMs for COGs.* The developers of the COG database construct position-specific scoring matrices (PSSMs) for each COG and use them to identify COG members within genomes [6, 7]. However, these models are not available in the publicly accessible COG database. Until recently, the NCBI CDD database [14] exclusively contained only models from the initial COG database version, COG 1.0. At present, this database lacks the COGs from the 2024 update, with COGs previously excluded by the authors, however, still present (as of September 1, 2025, the CDD database contains 5,137 PSSM models). Consequently, our prior research required a reconstruction of mathematical models for each COG based on the protein lists associated with them. At that stage, we decided to use hidden Markov models (HMMs), which are used, for example, in the Pfam protein domain database [15]. The methodology for constructing a profile HMM for each COG is comprehensively described elsewhere [12], and in summary, it included the following steps. First, proteins for each COG that are unique to that COG and are found in a taxonomically representative sample of genomes smaller than those in the COG database were selected. Second, multiple alignments of the protein sequences obtained by this selection process were created with Muscle 3.8.31 [16]. These alignments subsequently served as the basis for constructing profile HMMs using the HMMER 3.1b1 software package (http://hmmer.org/).

*Deriving profile HMMs for novel COGs.* We used the most recently published COG assignments for 1,309 and 2,296 prokaryotic genomes, selected by the COG database developers based primarily on: 1) the availability of a complete genome, and 2) the inclusion of one representative from each genus in the sample [7, 8]. For the 1,309 genomes, a sample of 210 genomes was chosen to be representative, and for the 2,296 genomes, 275 genomes were selected, see Tables S1 and S2 in the Supplementary materials for details. Consequently, our approach generally involved identifying a single representative organism from each taxonomic order, favoring those that were most extensively studied or served as model species. However, in cases of underrepresented taxa, such as archaea, we occasionally included multiple representatives. When a genome sample yielded fewer than ten protein identifications, we sought to use proteins from all genomes available in the corresponding COG database version. For some profile HMMs, version 3.1b2 of the HMMER package was used for technical reasons (as indicated in the file).

*Correcting profile HMMs.* An analysis revealed that the problems with multiple alignments were the predominant cause for the lower sensitivity of previously constructed profile HMMs. A significant number of these alignments underwent a manual analysis and subsequent reconstruction. We could not determine an accurate multiple alignment for representative sequences of several COGs despite manual examination, and some of them were subsequently removed from the COG database in 2020 (specifically, COG5022, COG5049, COG5074, COG5096, COG5183, COG5244, COG5354, COG5384, COG5602, and COG5624). We excluded the N- and C-terminal regions where the amino acid occurrence rate was less than 50% compared to the gaps from the remaining alignments. Furthermore, we removed proteins whose lengths remained substantially greater than the average sequence length within the alignment even after this exclusion procedure. Following this, our analysis focused on 332 alignments where the number of positions, despite the application of both methodologies described above, remained substantially larger than the upper quartile of the length distribution for the proteins involved in the alignment. For these specific cases, we aligned separate protein regions assigned to the corresponding COGs, rather than the complete protein sequences. Mostly, that was sufficient to ensure that the alignment length was no longer abnormally high, and the 23 alignments were analyzed manually on a case-bycase basis.

*Comparison of the constructed profile HMMs with other methods for mapping proteins to COGs.* In order to evaluate the quality of the protein assignment to COGs using our profile HMMs, we created two genome samples from the 2,296 genomes included in the latest version of the COG database. The first sample comprised 10 genomes from well-studied organisms, including model prokaryotes. Conversely, the second sample consisted of 10 genomes from poorly characterized organisms. Each subset contained five archaeal and five bacterial genomes (a detailed list of the selected genomes is provided in the Supplementary materials). Proteins from each genome in both samples were assigned to COGs using the eggNOG-mapper server (http://eggnog-mapper.embl.de/), using default parameters, including an e-value threshold of 0.001. For our profile HMMs, COG assignments were performed using a slightly more stringent e-value threshold of 0.0001, whilst the reference assignments were retrieved directly from the COG database. We compared protein sets classified as belonging to at least one COG via the aforementioned methods through visualization by the supervenn software (https://github.com/gecko984/supervenn).

*Availability of profile HMMs.* All constructed profile HMMs are available for a free download, both as a single file at https://boabio.belozersky.msu.ru/en/tools and individually for each COG. The set of profile HMMs presents a novel method for identifying protein regions homologous to COGs, differing from CDD database PSSMs both in the mathematical framework and multiple proteins alignments used. In total, manual analysis of an alignment was performed for 155 of the constructed profile HMMs.

**Eukaryotic genome selection**

The COG database, starting with the 2014 version, does not include eukaryotic genomes. For a general evaluation of whether sequences homologous to COGs are present in eukaryotes and in order to compare scores of eukaryotic findings with prokaryotic ones, we compiled a dataset of 102 eukaryotic genomes with predicted genes from diverse taxonomic lineages (for a complete list refer to Table S3 in the Supplementary material). The prior version of COGcollator included a dataset comprising 27 eukaryotic genomes, which is much less both by taxonomic diversity and volume. In numerous instances where organelle genomes were absent from the eukaryotic genome assembly, we performed a manual search and inclusion of these genomes, specifically incorporating 36 mitochondrial and five chloroplast genomes. Gene prediction within these subsequently added genomes was performed using Mfannot [17] where necessary.

**Acquiring source data for COGcollator**

We performed a search using the constructed profile HMMs via the hmmsearch program across three datasets: proteins assigned to COGs, proteins that could not be assigned to COGs, and proteins from the selection of 102 eukaryotes. For the current 2024 release of the COG database, the latest version of HMMER (v3.4) was used, whereas for the previous 2020 release, version 3.1b2 was employed.

**The DomainAnalyser tool for searching for profile HMMs**

The DomainAnalyser web server (https://boabio.belozersky.msu.ru/en/DomainAnalyser) enables the detection and visualization of profile HMM hits for COGs within any user-defined set of proteins. A detailed description of each parameter is provided on the website of the tool, with the interface available in both the Russian (default) and English languages.

**Obtaining novel COG assignments**

In the latest iteration of the COG database, which encompasses 2,296 genomes, 5.6 million sequences are assigned to COGs, with around 2.15 million sequences remaining unassigned to COGs. To assign protein regions to COGs, we applied the homology prediction procedure by the DomainAnalyser tool, which is specifically designed for this purpose. By employing a stringent e-value threshold (1e-15 or 10-15) alongside a rigorous overlap threshold of 5%, we successfully assigned over 180,000 additional proteins to COGs.

## RESULTS

**Overview of the COGcollator interface in the newest version**

**Fig. 2:**
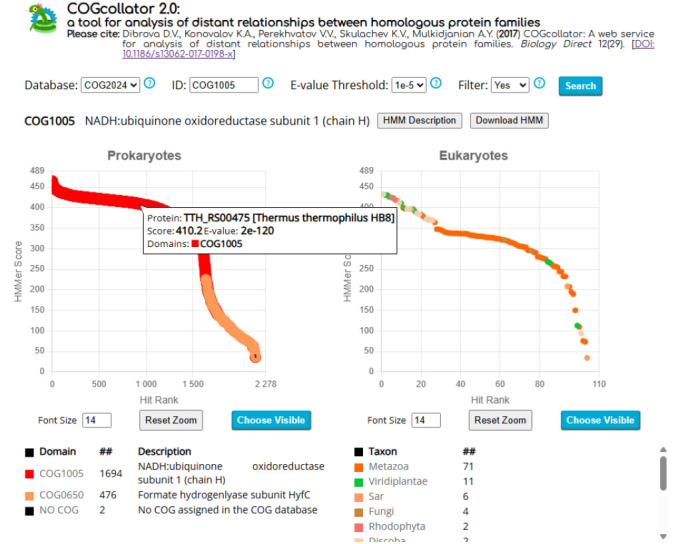
COGcollator interface showing the decrease in the HMMER score for the selected COG1005 as a function of the hit rank

The COGcollator tool (https://boabio.belozersky.msu. ru/en/COGcollator) displays two graphs for each COG, with the left one corresponding to prokaryotic hits and the right one corresponding to eukaryotic hits (Fig. 2). Each graph illustrates how the total alignment score of a hit decreases as the hit rank increases. These graphs offer interactive features, including zoom functionality, the retrieval of protein sequences from a currently visible set of dots, and the option to hide data based on COG or taxon for prokaryotic and eukaryotic graphs, respectively.

A user of the novel version of COGcollator may request information for a COG without a profile HMM. While the previous version would generate an input error, the new version details the cause of the profile HMM absence. Therefore, every profile HMM for a COG is provided with a summary detailing the procedure for its development.

**Additional functionality of COGcollator 2.0**

**Fig. 3:**
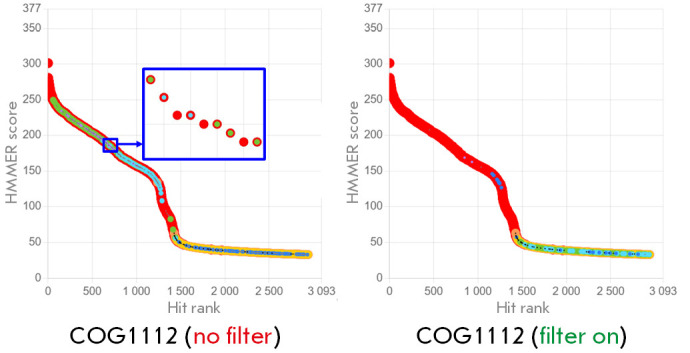
COGcollator plots for COG1112 (superfamily I DNA and/or RNA helicase) without hit filtering (left; the inset in the blue box shows an enlarged region containing fusions between COG1112, colored red, and either COG2251, colored blue, or COG2852, colored green) and with hit filtering enabled (right)

*The “Filter” option.* A mere occurrence of a COG in the resulting graph of the COGcollator does not inherently signify its homology to the query: it may coexist with the query in a fusion protein. Thus, the COGcollator outputs can be challenging to interpret when analyzing COGs that are frequently involved in fusions, such as the subunits of polyketide synthase complexes homologous to fatty acid synthases, or proteins of two-component regulatory systems. By enabling the “Filter” option, users can hide COG assignments that do not overlap by coordinates with the current profile HMM hit (Fig. 3).

*Optional e-value threshold.* In the previous version of COGcollator, each profile HMM was represented by a single graph, truncated at the alignment score corresponding to an e-value threshold of 10-5. The new version enables data calculation for each COG at an e-value threshold of 0.1, allowing for the observation of more distant results.

**Comparison of the performance of our profile HMMs with alternative tools**

The search performance of a specific set of profile HMMs or other models for protein families can be roughly estimated through the total number of proteins successfully assigned to at least one family. According to this criterion, our set of profile HMMs for COGs identified 19,867 out of 25,273 protein sequences encoded within the dataset of 10 well-studied organism genomes. In comparison, the original version of the COG database identified 18,611, while the eggNOG COG annotation results yielded either 21,301 proteins (if archaeal-specific COGs, or arCOGs, are included) or 19,036 proteins (excluding them) (Fig. S1A in the Supplementary material). For a sample of genomes from comparatively understudied organisms, encompassing a total of 24,251 proteins, the corresponding hit counts were 18,764, 16,680, 14,938, and 14,672 (Fig. S1B, Supplementary material). These data indicate that our set of profile HMMs assigned an equal, and for less-studied organisms even greater, number of proteins to COGs. A more detailed comparison of the protein sets identified by these different methods is provided in the caption to Fig. S1 in the Supplementary material.

We were also satisfied with the proportion of members of each COG that were not identified by our profile HMMs relative to the total number of members (Table S4 in the Supplementary material). Specifically, for 95% of the profile HMMs, the proportion of proteins not identified did not exceed 5%, and for only 0.6% did it exceed 20% (in such cases, we offer an explanation).

## DISCUSSION

**Fig. 4:**
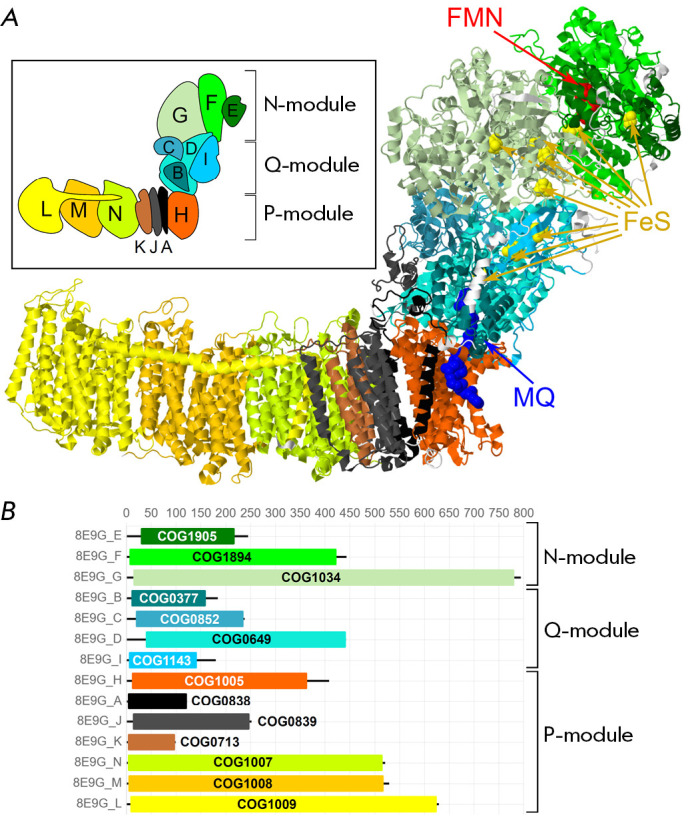
Structural organization of the NADH:quionone oxidoreductase (NDH-1) from Mycobacterim smegmatis MC2 155 [26] (PDB ID: 8E9G) and the assignment of the corresponding proteins from COGs according to DomainAnalyser (https://boabio.belozersky.msu.ru/ en/DomainAnalyser). (A) Overall structure of the NDH-1 complex; part of the complex on the n-side of the membrane is shown on top. Colored regions in proteins depict the corresponding regions which are assigned to COGs by DomainAnalyser. (B) Hits of COGs profile HMMs for the NDH-1 subunits depicted through the DomainAnalyser web server. Colors correspond to panel (A)

The COGcollator and DomainAnalyser tools provide a user-friendly platform for analyzing both individual proteins and protein complexes utilizing the COG database. We used these tools to search for homologues of the subunits of NADH:quinone oxidoreductase type 1 (NDH-1), the prokaryotic homologue of mitochondrial complex I [18, 19, 20]. Our results can be contextualized through a comparison with the previously established general evolutionary history of NDH-1 [21, 22, 23, 24, 25]. This enzyme is composed of three functional modules (Fig. 4).

**Fig. 5:**
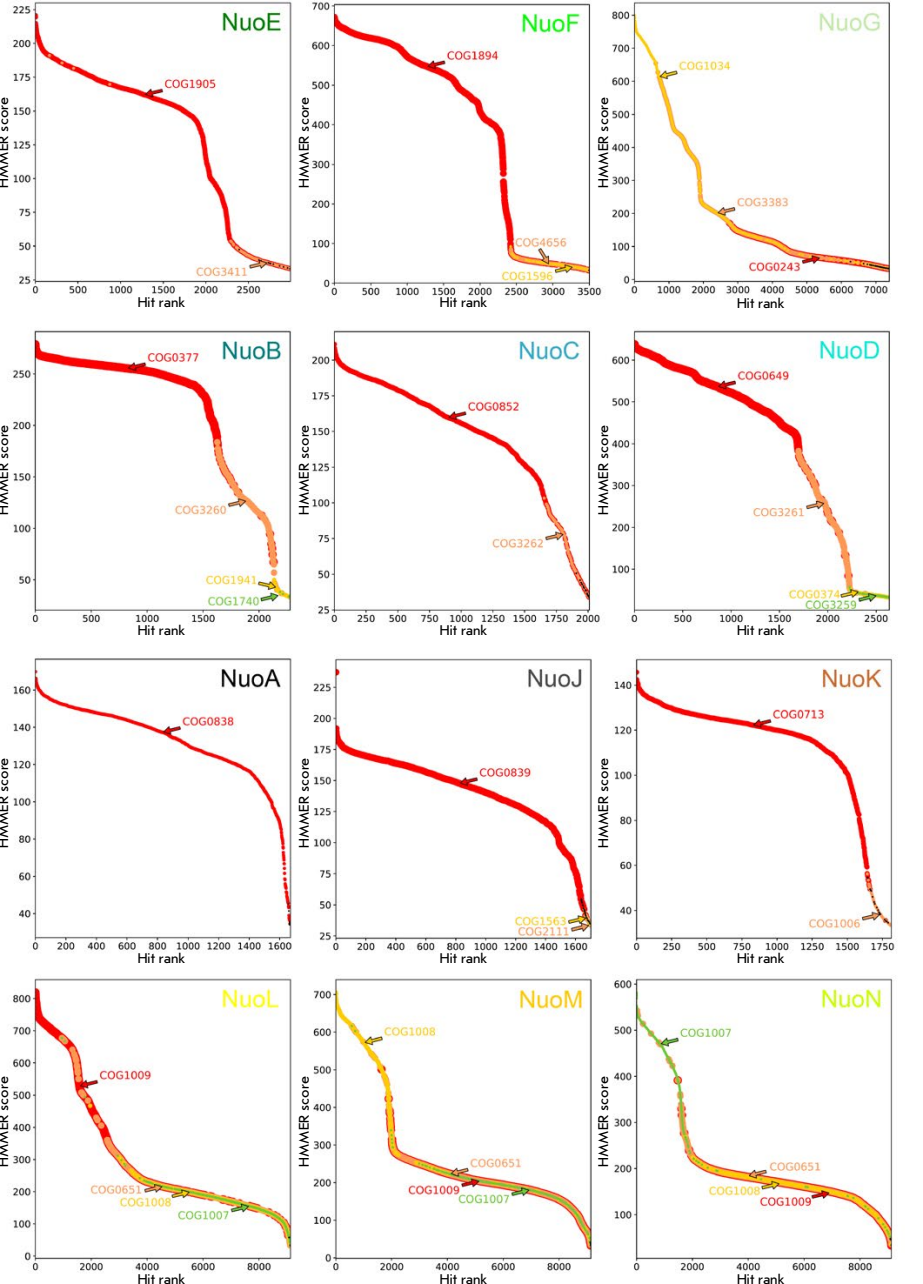
An example of the COGcollator outputs for the prokaryotic hits of the main subunits of NDH-1. The graph for the NuoI subunit (COG1143) is not shown, and the COGcollator graph for NuoH (COG1005) is shown in Fig. 2. Filtration of obvious fusions is turned on, and an e-value threshold of 1e-5 is used

The subunits of the N-module catalyze the oxidation of NADH and electron transfer along the chain from flavin mononucleotide (FMN, shown in red in Fig. 4) and iron–sulfur clusters (FeS, shown in yellow in Fig. 4; the FeS cluster marked with a dashed arrow does not participate in electron transfer and is absent from many homologous complexes) to the Q-module. The Q-module subunits also contain iron– sulfur clusters and direct electrons through them to the quinone-binding site (which, in the M. smegmatis complex, binds menaquinone [MQ], shown in blue). This site is located between the NuoD subunit and the transmembrane NuoH subunit. The membraneembedded arm of the complex (the P-module) is responsible for the energy-consuming translocation of protons from the n-side to the p-side of the membrane (this process is coupled with quinone reduction). The COGcollator data for the majority of the NDH-1 subunits are presented in Fig. 5.

As expected, the subunits of the N-module found similarity among other proteins containing iron–sulfur clusters (Fig. 5, top row). The COGcollator suggests COG3411 (ferredoxin with a Fe2 S2 iron-sulfur cluster) to be homologous to NuoE (COG1905). This fully corroborate the findings previously derived from sequence [27] and structural [28] comparisons. The evolutionary relationship between COG1894 (NuoF) and COG4656 (the RnfC subunit of Rnf complex) was previously inferred exclusively from structural similarities [29, 30]; however, a sequence-based COGcollator also supports it. The second COG (COG1596) detected in the graph is an artifact: the members of this COG typically contain several tandemly repeating segments that are detected with a low score by the profile HMM for COG1894, but due to the large number of such segments, the overall score of the match proves to be significant. This type of artifact can be identified using DomainAnalyser (see Fig. S2 in the Supplementary material). Furthermore, the COGcollator analysis for COG1596 does not indicate any COG1894 relatedness (Fig. S3A in the Supplementary material), in contrast to the COGcollator results for genuinely homologous COG4656 (Fig. S3B in the Supplementary material). The COGcollator analysis for NuoG (COG1034) yielded predictable outcomes, reinforcing the documented relationship between this subunit and both molybdopterin-containing and iron-containing dehydrogenases [21, 23]. Specifically, the results include COG3383, which functions as the catalytic subunit of formate dehydrogenase ForCE, and COG0243, annotated as a putative anaerobic dehydrogenase. The COGcollator graph for NuoG serves to highlight the fact that red is not always the first color (from left to right) in the graph: colors are assigned sequentially based on the number of hits for each COG among the proteins displayed on the graph. In this case, the number of hits attributed to COG0243 and COG3383 exceeded the number of hits attributed to COG1034.

The COGcollator graphs for the Q-module subunits (the second row in Fig. 5), NuoB (COG0377), NuoC (COG0862), and NuoD (COG0649), demonstrate their similarity to the subunits of type III Ni–Fe hydrogenase: the small subunit (COG3260), G component (COG3262), and the large subunit (COG3261), respectively. The NuoB and NuoD subunits demonstrate a considerably more remote similarity, on the one hand, to the subunits of the coenzyme F420-reducing hydrogenase, specifically the γ-subunit (COG1941) and the α-subunit (COG3259), and, on the other hand, to the subunits of water-soluble type I Ni-Fe hydrogenases, specifically the small subunit (COG1740) and the large subunit (COG0374). Consequently, the results of the Q-module analysis using COGcollator corroborate the hypothesis that the NuoB, NuoC, and NuoD subunits constitute a core cytoplasmic module that links NDH-1 with membrane-bound hydrogenases [24]. Drawing definitive conclusions regarding the NuoI subunit and its membrane hydrogenase homologue remains challenging due to the presence of numerous other proteins within this COG. The current COG database version registers 1,717 NuoD homologues and 1,601 NuoB homologues. Conversely, COG1143, which corresponds to NuoI, includes 4,813 proteins, a quantity more than twice as large.

It is a well-established observation that certain subunits of the NDH-1 P-module exhibit homology with subunits of membrane hydrogenases, whereas others are homologous to subunits of Na^+^/H^+^ antiporters belonging to the Mrp family. Furthermore, it has been demonstrated that some membrane hydrogenases incorporate components from Mrp antiporters [21, 23, 31, 32]. This finding is further substantiated by the COGcollator plots for the P-module subunits (the third and fourth rows in Fig. 5, and the plot on the left in Fig. 2). The evolutionary link between the MnhA subunit of Mrp antiporters and the NuoL subunit is so close that they are grouped within a single COG. Starting from 2024, a third protein, MpsA (a NuoL homologue that is likely capable of transporting sodium ions), has also been annotated within this COG [33,34, 35]. The mpsA gene is present within the genomic sequences of numerous prokaryotic organisms, including pathogenic species like Staphylococcus aureus, where it colocalizes adjacent to the gene responsible for a large cytoplasmic subunit associated with COG3002 [36]. The COGcollator graphs illustrate a close similarity between the main subunits of the NuoL P-module (COG1009), NuoM (COG1008), and NuoN (COG1007). This analysis also reveals their similarity to the Mrp antiporter subunit MnhD (COG0651), which is likewise present in specific membrane hydrogenases. The NuoH subunit (COG1005), involved in the quinone reduction process, possesses a single related subunit: the transmembrane subunit of membrane hydrogenases, belonging to COG0650 (refer to Fig. 2). The COGcollator graphs for two of the three small P-module subunits indicate a distant relationship with the small subunits of Mrp antiporters: NuoJ (COG0839) is found to be similar to MnhB (COG2111) and its related protein (COG1563), whilst NuoK (COG0713) proves to resemble MnhC (COG1006). No proteins related to the NuoA subunit (COG0838) were detected, and it is worth mentioning that a gene encoding NuoA is positioned at the very beginning of the typical NDH-1 operon, detached from the other P-module subunits.

## CONCLUSIONS

The statistical significance of the evolutionary relationships between COGs identified using COGcollator depends directly on the following: 1) the alignment score (and corresponding e-value) for hit detection; 2) whether the protein region containing the hit significantly overlaps with the region assigned to the COG (the new version of COGcollator allows one to filter out non-overlapping hits); and 3) the abundance of hits from a given COG visible on the plot, as single COG assignments may simply be erroneous and are, thus, insufficient to establish homology. It is also worth checking whether the presumed evolutionary relationship is detected when the identified COG is used as a query, as discussed above using the example of NuoF.

The analysis of the NDH-1 enzyme complex demonstrates that the COGcollator tool provides reliable data regarding the relationships between enzyme families. The tool presents data clearly and allows for an individual analysis of each protein through its interactive functions and the complementary DomainAnalyser tool. For example, using COGcollator, we identified proteins homologous to components of the Rnf enzymatic complex (see Figs. S4 and S5 in the Supplementary material) which were found to be entirely consistent with previously published data [37, 38].

## Abbreviations

COG: Cluster of Orthologous Groups
HMM: Hidden Markov Model
PSSM: PositionSpecific Scoring Matrix
NDH-1: NADH:quinone oxidoreductase type 1
